# Focused helium-ion beam irradiation effects on electrical transport properties of few-layer WSe_2_: enabling nanoscale direct write homo-junctions

**DOI:** 10.1038/srep27276

**Published:** 2016-06-06

**Authors:** Michael G. Stanford, Pushpa Raj Pudasaini, Alex Belianinov, Nicholas Cross, Joo Hyon Noh, Michael R. Koehler, David G. Mandrus, Gerd Duscher, Adam J. Rondinone, Ilia N. Ivanov, T. Zac Ward, Philip D. Rack

**Affiliations:** 1 Department of Materials Science and Engineering, University of Tennessee, Knoxville, Tennessee 37996, USA; 2 Center for Nanophase Materials Sciences, Oak Ridge National Laboratory, Oak Ridge, Tennessee 37831, USA; 3 Materials Science and Technology Division, Oak Ridge National Laboratory, Oak Ridge, Tennessee 37831, USA

## Abstract

Atomically thin transition metal dichalcogenides (TMDs) are currently receiving significant attention due to their promising opto-electronic properties. Tuning optical and electrical properties of mono and few-layer TMDs, such as tungsten diselenide (WSe_2_), by controlling the defects, is an intriguing opportunity to synthesize next generation two dimensional material opto-electronic devices. Here, we report the effects of focused helium ion beam irradiation on the structural, optical and electrical properties of few-layer WSe_2_, via high resolution scanning transmission electron microscopy, Raman spectroscopy, and electrical transport measurements. By controlling the ion irradiation dose, we selectively introduce precise defects in few-layer WSe_2_ thereby locally tuning the resistivity and transport properties of the material. Hole transport in the few layer WSe_2_ is degraded more severely relative to electron transport after helium ion irradiation. Furthermore, by selectively exposing material with the ion beam, we demonstrate a simple yet highly tunable method to create lateral homo-junctions in few layer WSe_2_ flakes, which constitutes an important advance towards two dimensional opto-electronic devices.

Two-dimensional transition-metal dichalcogenides (TMDs) have recently garnered interest due to their novel electronic and optoelectronic properties and provide promise for next generation device technologies. TMDs belong to the MX_2_ family where M = W, Mo, or Nb and X = Se, S, or Te^1^,^2^. Much of the interest in TMDs is fueled by the presence of a band gap, which enables the creation of atomically thin semiconductor devices that are otherwise difficult to fabricate from intrinsically gapless materials such as graphene.

Single layer WSe_2_ has a direct band gap of ~1.67 eV^3^ and an indirect band gap of ~1.2 eV^4^ in the bulk, which is in the visible spectrum. High quality WSe_2_ films can be easily fabricated by mechanical exfoliation from single crystal down to a single, or a few layers. Exfoliated WSe_2_ layers have been successfully used in thin-film transistors^5^, electrostatically gated light emitting diodes^6^,^7^, and electrostatically gated photodiodes^8^ to name a few. Chemical vapor deposition (CVD) growth has been used to create large area synthesis of TMD monolayers^9^ as well as lateral heterojunctions between TMDs of different composition^10^. This advance has allowed the realization of devices with precisely controlled thicknesses to be functionalized by lateral junctions^10^,^11^.

Tuning of defects within TMD devices serves as an alternative method to vary electronic and optoelectronic properties. Irradiation with charged particle beams allows precise control of defect generation by altering beam conditions and exposure dose. Kim *et al.* demonstrated the use of a high energy proton beam to introduce trap states in the back gate dielectric of a MoS_2_ thin-film transistor^12^. Tongay *et al.* have used α-particle irradiation to generate vacancies in TMDs, which introduce new emission peaks and enhance photoluminescence intensity^13^. Fox *et al.* have demonstrated the use of a focused helium-ion beam to pattern MoS_2_ as well as preferentially sputter sulfur atoms^14^. The local tuning of opto-electronic properties of mono and few-layer TMDs can provide an excellent opportunity to realize sharp homo-junctions similar to conventional p-n, p-i-n, or p-n-p junctions, which are critical to many device architectures. The p-n junction diodes are particularly important because the built-in potential at the junction separates the photo-generated electron-hole pairs, which subsequently migrate to the respective electrodes, leading to higher photo-current at zero bias. Both vertical and lateral, homo- and hetero- p-n junctions have been realized in many TMDs by chemical doping^15^,^16^, electrostatic doping^6^,^7^,^8^, and material engineering^10^,^17^,^18^. However, chemical doping may require capping layers, or additional lithographic steps, adding complexity to device fabrication, whereas the electrostatic doping brings many challenges for nanoscale modification.

In this study, we selectively introduced defects in few-layer WSe_2_, including chalcogen vacancies, by irradiation with a focused He^+^ beam. Signatures of induced disorder are apparent in the measured electronic and optoelectronic properties. Specifically, He^+^ irradiation of WSe_2_ causes a semiconductor – insulator – metallic transition with increasing dose due to induced disorder and preferential sputtering of selenium atoms. Ambipolar conduction of WSe_2_ transistors is quenched at an exposure dose of 1 × 10^15^ He^+^/cm^2^, thus the defects generated by He^+^ exposure effectively act as a highly tunable method to direct write n-type dopants. We have demonstrated selective He^+^ irradiation within a few-layer WSe_2_ flake as a novel method to introduce an optically active homo-junction, similar to a conventional p-n junction.

## Results

**Figure 1:**
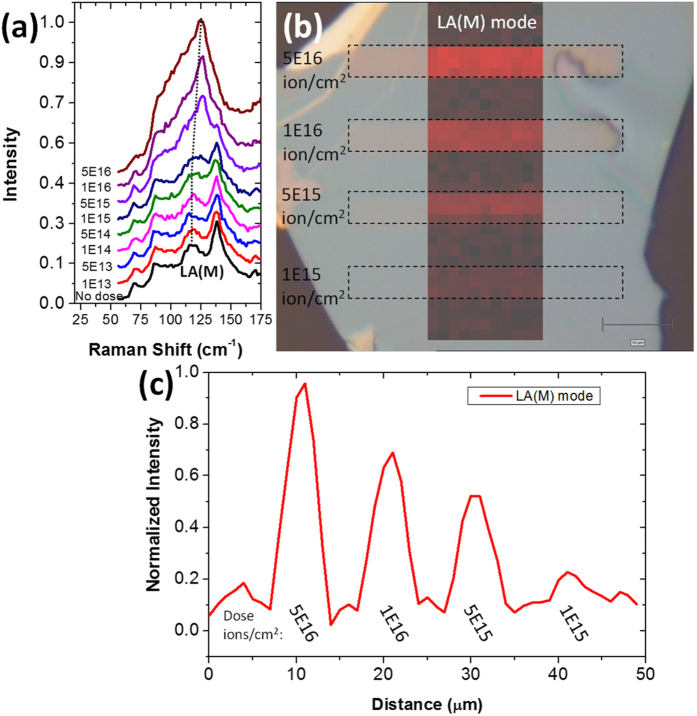
(**a**) Raman spectra of WSe_2_ showing the LA(M) peak at ~118 cm^−1^. (**b**) Spatially resolved Raman map of the LA(M) peak superimposed onto an optical micrograph. Rectangular He^+^ exposures on the flake are denoted by inset dotted lines. (**c**) Normalized intensity of LA(M) mode along a line scan on the patterned WSe_2_ flake.

Figure 1a illustrates Raman spectra for exfoliated few-layer WSe_2_. The longitudinal acoustic (LA) mode at the M point of the Brillouin zone (LA(M)) is particularly interesting as this peak is associated with defect generation and disorder within the lattice^19^,^20^, analogous to the D band in graphene. As the He^+^ irradiation dose increases, the intensity of the LA(M) peak increases and also shifts from ~118 cm^−1^ to 124 cm^−1^, thus indicating defect generation in the WSe_2_. A spatially resolved Raman map of the LA(M) peak intensity is shown superimposed on an optical micrograph in Fig. 1b. The rise in intensity of the LA(M) peak confirms direct-write defect generation in WSe_2_ by He^+^ irradiation. Figure 1c is a line plot of the LA(M) peak intensity across the WSe_2_ flake. The intensity of the LA(M) peak correlates with the irradiation dose and indicates that greater He^+^ doses introduces greater disorder within the flake. Due to resolution limits of micro-Raman, the generated exposure patterns were large (>4 μm) relative to the resolution limits of the He^+^ microscope (<1 nm). Thus, direct-write defect generation on the nanoscale is straightforward, though proximal disorder from the backscattered ion beam, in the case of supported samples, must be considered^21^,^22^. Additional Raman spectra and peak assignments may be found in the Supplementary Information.

**Figure 2:**
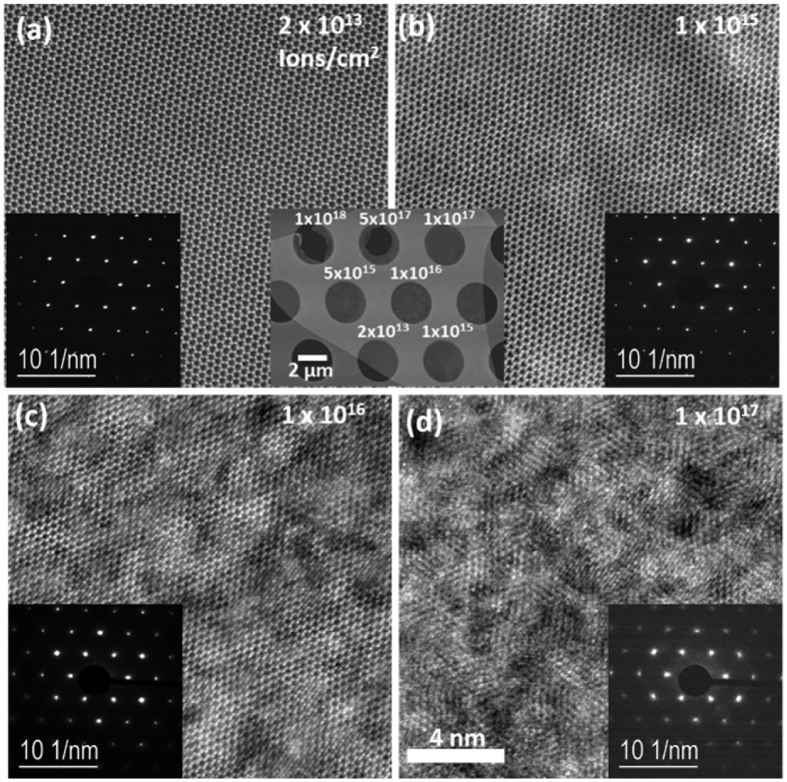
HAADF STEM images of suspended WSe_2_ which was irradiated with He^+^ at doses of (**a**) 2 × 10^13^, (**b**) 1 × 10^15^, (**c**) 1 × 10^16^, and (**d**) 1 × 10^17 ^ions/cm^2^. Field of view is 16 nm. SAED patterns are inset for each exposed dose.

Figure 2 shows HAADF STEM images of suspended few-layer WSe_2_ irradiated with various doses of He^+^. Figure 2a, shows that a dose of 2 × 10^13 ^ions/cm^2^ has little effect on the single crystal structure of the exfoliated WSe_2_, which appears relatively free of point defects. As the dose increases to 1 × 10^15^ and 1 × 10^16 ^ions/cm^2^ in Fig. 2b,c, signs of disorder in the film become evident and result in a semi-crystalline WSe_2_ film. When the dose is increased to 1 × 10^17 ^ions/cm^2^ (Fig. 2d), the films becomes significantly disordered. Additional STEM images can be found in the Supplementary Information. Inset SAED patterns show broadening of diffraction spots with increasing He^+^ dose, however the position of the distinct spots did not change indicating that the overall orientation and phase of the WSe_2_ did not change. Results from Z-contrast imaging and SAED both show a trend towards increasing disorder of the lattice structure as ion dosage is increased. This disorder can be considered an increased amount of point defects, consistent with the Raman spectra. However, only the 1 × 10^17 ^He^+^/cm^2^ SAED pattern has significant contributions from random scattering events and broadened Bragg spots, which suggest increased disorder in the crystalline periodicity. This indicates a significant change in crystal structure with a 1 × 10^17 ^He^+^/cm^2^ exposure dose. This is likely due to increasing selenium vacancies degrading short-range order in the material, which is confirmed by EDS chemical composition analysis detailed in Supplementary Information, and agrees with previous work^14^. It is worth noting that backscattered He^+^ will have a negligible effect on the defect generation in the suspended WSe_2_, since the ions pass through the film and into vacuum. In contrast, substrate-supported WSe_2_ will experience collisions from backscattered ions, which expedites the formation of the point defects.

**Figure 3:**
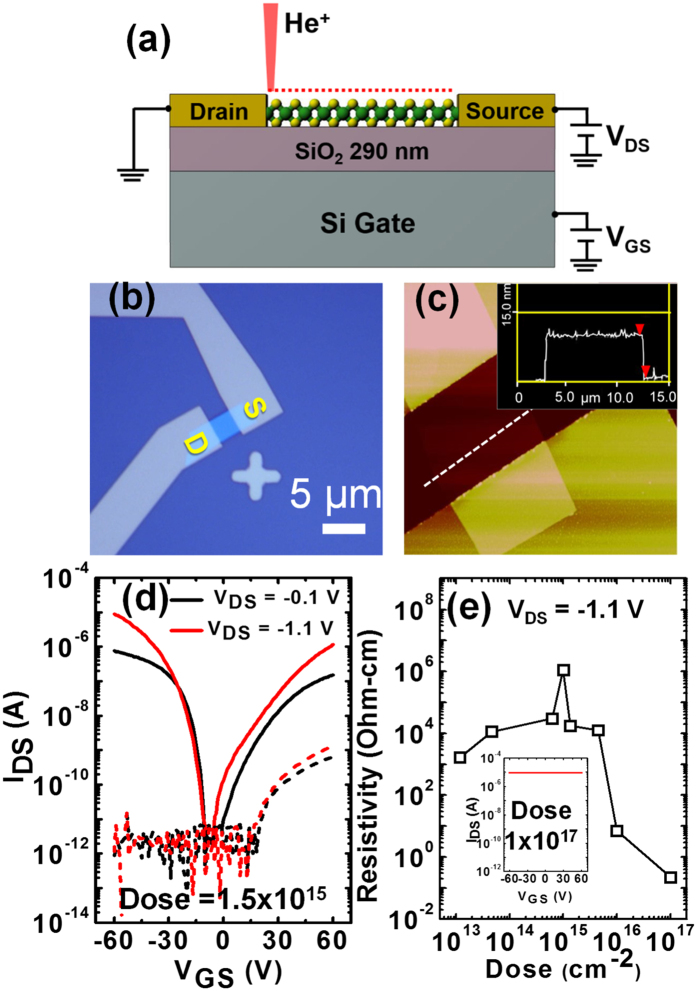
(**a**) Schematic of the WSe_2_ field effect transistor (FET) device irradiated with He^+^. (**b**) Optical micrograph of the WSe_2_ FET on SiO_2_/Si substrate. The scale bar is 10 μm. (**c**) AFM micrograph of the fabricated WSe_2_ FET device. The inset represents the height profile along the dotted line shown in the figure. (**d**) The transfer characteristics (I_DS_ vs V_GS_) at two different drain-source voltages (black curves for V_DS_ = −0.1 V and red curves for V_DS_ = −1.1 V) before (solid curves) and after (dashed curves) He^+^ irradiation at a dose of 1.5 × 10^15 ^ions/cm^2^ on WSe_2_ channel region. The measured transfer characteristic clearly shows the ambipolar characteristics of the WSe_2_ FET device before He^+^ irradiation, while the device after He^+^ irradiation loses its p-type characteristics. (**e**) Double log plot of electrical resistivity as a function of He^+^ irradiation dose for mechanically exfoliated few layers WSe_2_ flakes on SiO_2_/Si substrate. Gradually insulating behavior arose with the initial increasing dose applied, while metallic behavior was observed with the further increase in dose applied. The gate tunability of the WSe2 device was completely reduced as seen in inset, at a dose of 1 × 10^17 ^ions/cm^2^.

The effects of He^+^ irradiation on electrical transport properties of mechanically exfoliated few-layer WSe_2_ films were studied using a field effect transistor (FET) configuration. Figure 3a shows a schematic of a WSe_2_ FET device on SiO_2_/Si substrate with symmetric Ti/Au contacts. Figure 3b is an optical image of a fabricated device. The AFM micrograph and height profile of one of the devices are shown in Fig. 3c. Few-layer WSe_2_ FET devices with flake thicknesses ranging from 7–26 nm were used in this study. The devices were irradiated with different He^+^ doses ranging from 1 × 10^13^ to 1 × 10^17 ^He^+^/cm^2^ with the Zeiss ORION NanoFab He/Ne ion microscope with the beam conditions as described in the experimental section. Figure 3d shows the transfer curves at two different drain-source voltages (black curves for V_DS_ = −0.1 V and red curves for V_DS_ = −1.1 V) before (solid curves) and after (dotted curves) the He^+^ irradiation at a dose of 1.5 × 10^15 ^ions/cm^2^. The measured transfer characteristics clearly show the ambipolar characteristics of the WSe_2_ FET device prior to He^+^ irradiation, consistent with previous reports^23^,^24^. The source-drain current (I_DS_) increases with an increase in V_GS_ both with positive and negative bias almost symmetrically reaching an ON state current >1 μA, leading the current ON/OFF ratio in excess of 10^6^. The device after He^+^ irradiation (dotted curves) has a degraded hole conduction with a six orders of magnitude decrease in ON state current at negative gate bias, while the ON state current for the positive gate bias (electron conduction) decreases by approximately three orders of magnitude. For clarity, a single voltage sweep of the transfer curve (I_DS_ vs V_GS_) was plotted, however we observed a small hysteresis both before and after He^+^ irradiation in WSe_2_ FET device (see Supplementary Information file Fig. S7). The field effect mobility of the pristine device (prior to He^+^ exposure) shows thickness dependence which agrees with the literature^1^. The maximum field effect hole mobility of 64.13 cm^2^/Vs was determined for a 9 nm thick device (see Supplementary Information Fig. S8 for thickness dependent mobility). The field effect hole mobility of the same device after He^+^ ion irradiation was almost negligible (0.0052 cm^2^/V.s), while there is still small electron conduction. Furthermore, He^+^ irradiation effects as a function of WSe_2_ film thickness was also studied at a particular dose of 1 × 10^15 ^ions/cm^2^. I-V measurements reveal that irrespective to the WSe_2_ channel thickness, both hole and electron conductivity were suppressed. However, hole conduction decreased more than electron conduction, and it shows slightly n-type behavior (increase in channel current with the increase in gate voltage) (see Supplementary Information file Fig. S9). The 25 keV He^+^ used in this study is very energetic and easily penetrates the entire thickness of the few-layer WSe_2_ channel. We performed EnvizION ion-solid Monte Carlo simulations illustrating the distribution of displaced atoms in WSe_2_ films of varying thickness (see Supplementary Information file Fig. S10). The thickest few layer films we tested were 26 nm thick and thus significantly thinner than the He^+^ penetration depth which has a peak implant depth of ~120 nm in bulk WSe_2_. While the energy is slightly dissipated and thus the electronic and nuclear stopping power slightly changed from top to bottom, it is negligible and thus we expect a fairly uniform defect distribution within the material.

Figure 3e is a plot of the resistivity evolution of the mechanically exfoliated few-layer WSe_2_ on SiO_2_/Si supported architecture as a function of He^+^ dose. We observe three distinct regimes as a function of the He^+^ dose. The initial semiconducting nature of the material changes to insulating behavior with a two order of magnitude increase in resistivity at the He^+^ dose of ~1 × 10^14 ^ions/cm^2^ and more than four orders of magnitude increase in resistivity at 1 × 10^15 ^ions/cm^2^. As the dose increases, the resistivity of the device decreases sharply and reaches approximately two orders magnitude lower resistivity than the initial pristine device. At the highest dose, (1 × 10^17 ^ions/cm^2^) the WSe_2_ device completely loses its semiconducting behavior (see the inset in Fig. 3e) as the current is no longer sensitive to the gate voltage. Similar semiconductor-insulator-metal transitions have been previously reported for MoS_2_ layered materials with the He^+^ exposure^14^. An increase in electrical resistivity in layered MoS_2_ due to high energy proton beam irradiation has also been reported^12^. The electrical resistivity changes of the proton-irradiated MoS_2_ was attributed to induced traps, including positive oxide-charge traps, in the underlying SiO_2_ gate insulator layer, and the trap states at the interface between the MoS_2_ channel and SiO_2_ layer. However, in contrast to proton irradiated MoS_2_ where the current recovered almost to its original values after five days, our He^+^ irradiated WSe_2_ devices do not recover even after a month (see Supplementary Information file Fig. S11).

The observed electrical changes due to the He^+^ irradiation can be understood by considering the structural changes in the few-layer WSe_2_ under the He^+^ irradiation. EDS analysis (Fig. S5) and a previous study^14^, show that He^+^ irradiation results in the preferential sputtering of chalcogen atoms. Density Functional Theory calculations suggests that chalcogen vacancies in TMDs result in unsaturated electrons which surround the transition metal atoms and act as electron donors^25^. In the case of MoS_2_, S vacancies act as deep donor states. These states demonstrate high electron mass and strong localization within a 3 Å radius surrounding the vacancy. This results in a nearest-neighbor hopping transport mechanism at room temperature. This is in stark contrast to delocalized electrons in the valence band which are dominated by Mo 4d orbitals.

Analogously, Se vacancies in WSe_2_ act as electron donors, and thus an n-type dopant. When irradiated with relatively low He^+^ dose (1 × 10^13^–5 × 10^15 ^ions/cm^2^), Se vacancies are formed through knock-on collisions. These vacancies create highly localized states which serve as hole traps. This accounts for the reduced hole conduction in devices which were irradiated with He^+^. Since the near mid gap Se vacancy states are highly localized, electron conduction is not significantly enhanced by He^+^ irradiation and scattering at defect sites can explain the slight degradation in electron conduction and increased resistivity. Thus, direct-write introduction of Se vacancies through He^+^ irradiation serves as a method to selectively quench hole conduction while permitting electron conduction. At high He^+^ dose (>1 × 10^16 ^ions/cm^2^), selective Se sputtering greatly increases the W atomic percentage. This enables metallic bonding between neighboring W which increases electron delocalization, hence producing a large drop in electrical resistivity (Fig. 3e). It is worth noting, oxygen substitution into Se vacancy sites under room temperature ambient conditions may occur, but the rate is slow without supplying additional thermal energy^26^. We conclude that oxidation of Se vacancy sites does not play a dominant role in influencing the electrical behavior of He^+^ irradiated WSe_2_ flakes, since device behavior shows minimal changes with time, and Raman spectra do not show signatures of oxidation^27^.

**Figure 4:**
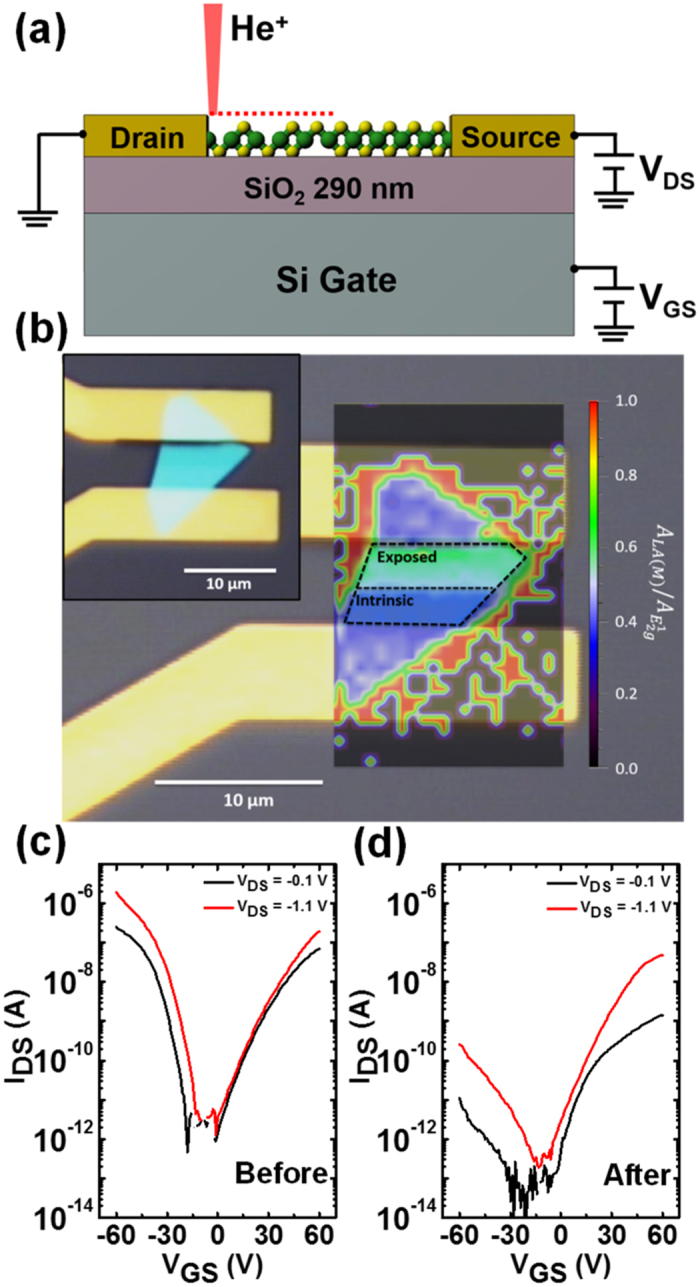
(**a**) Schematic of the WSe_2_ field effect transistor (FET) device irradiated with He^+^ over half of the channel length to induce a homo-junction. (**b**) Spatially resolved Raman map of He^+^ irradiated junction (1 × 10^15 ^ions/cm^2^) on a WSe_2_ flake. Map shows ratio of integrated peak area of LA(M) (associated with defects) to the main Raman peak E^1^_2g_. The inset in the upper left corner shows an optical micrograph of WSe_2_ device. The measured transfer characteristics of a WSe_2_ FET device (**c**) before and (**d**) after, He^+^ irradiation was used to create a homo-junction at a dose of 1 × 10^15 ^ions/cm^2^.

The selective suppression of hole transport in ambipolar WSe_2_ flakes due to He^+^ irradiation can generate a homo-junction similar to a conventional p-n junction. The ability to quickly create this structure in a simple, robust, and tunable manner is critical to realizing many opto-electronics devices. Therefore, we selectively irradiated half of the channel area of WSe_2_ FET devices. Figure 4a shows a schematic of an irradiated device, in which selective introduction of defects are used to create a homo-junction within the WSe_2_ flake. Figure 4b shows a spatially resolved Raman map of this device, which plots the integrated peak area ratio of the LA(M) peak (which is associated with He^+^ induced disorder) to the in-plane E^1^_2g_ main peak. It is clear that He^+^ irradiation successfully induced a junction within the material, as revealed by Raman, although the optical micrograph (see inset in Fig. 4b) shows no visual signature of the irradiation. The electrical transfer characteristic curves of the corresponding device, before and after He^+^ irradiation, are shown in Fig. 4c,d, respectively. Consistent with the previous observation, the hole transport in the material decreased by almost four orders in magnitude. For example, the device ON current was measured as 1.86 μA, at V_DS_ = −1.1 V and V_GS_ = −60 V, prior to He^+^ irradiation; whereas after He^+^ irradiation, the current was measured as 0.26 nA under the same measurement conditions. In contrast, the transistor ON current, corresponding to the electron transport in the device, decreases by less than an order of magnitude.

**Figure 5:**
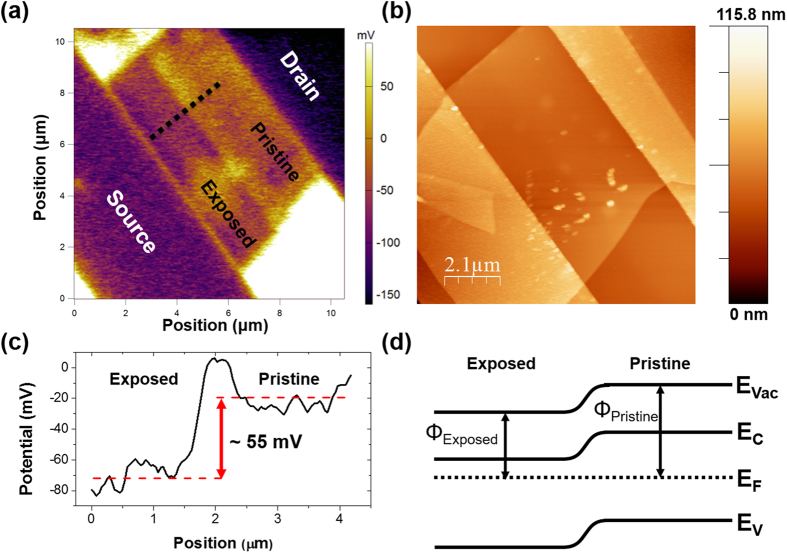
(**a**) Kelvin Probe Force Microscopy (KPFM) image of a WSe_2_ TFT with symmetric Ti/Au electrodes in which half of the channel with exposed with a dose of 5 × 10^14 ^He^+^/cm^2^. (**b**) Tapping mode AFM image of the same exposed WSe_2_ TFT, which shows no topographical evidence of exposure. (**c**) KPFM line scan, denoted by a black dotted line in (**a**), which shows band bending at the interface of exposed and pristine WSe_2_. (**d**) Band diagram of a WSe_2_ flake, which has a junction created by He^+^ exposure.

Figure 5a shows a Kelvin Probe Force Microscopy (KPFM) image of a WSe_2_ TFT with a homo-junction created within the flake by exposing half of the channel with a dose of 5 × 10^14 ^He^+^/cm^2^. The homo-junction is visible within the channel and the interface is sharp. The junction indicates clear band bending in the vacuum level and represents the difference in work function (Φ) of the exposed and pristine WSe_2_. It is worth noting that the scale of the surface potential is offset due to charging effects related to poor grounding of the device, therefore the magnitude of potential differences at the interfaces should be noted as opposed to the absolute magnitude. Figure 5b is a tapping mode topography AFM image of the same WSe_2_ device. The topography of the WSe_2_ flake shows no signs of surface alteration as a result of the He^+^ exposure. Hence, the structural integrity of the device remains intact, while the electronic structure is tuned by precisely controlling the exposure dose. Figure 5c illustrates a KPFM line scan, along the black dotted line in Fig. 5a, which shows band bending across the WSe_2_ homo-junction. The work function difference between the exposed and pristine region is ~55 mV for a junction created with a dose of 5 × 10^14 ^He^+^/cm^2^. Figure 5d depicts a proposed band diagram of the homo-junction created within the WSe_2_ device. The exposed region takes on n-type behavior, which limits hole transport, and is experimentally observed in the transport properties (Fig. 4d). The electrical properties and hence band bending at the homo-junction is tunable by controlling He^+^ dose, as indicated by changes in transport properties with dose (Fig. 3e).

**Figure 6:**
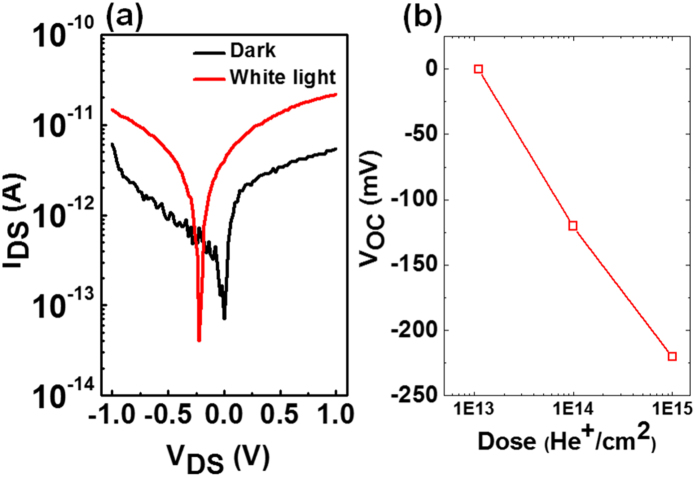
(**a**) The photoresponse of a device with the lateral homojunction created by a dose of 1 × 10^15 ^He^+^/cm^2^ in WSe_2_ at zero gate bias. The seimi-log plot of I_DS_ vs V_DS_ with and without light exposure shows the photoresponse with noticable photovoltage as high as 220 mV. (**b**) Open circuit voltage extracted from devices under light condition as a function of He^+^ dose used to create the homo-junction.

The photo-response of the lateral homo-junction created in layered WSe_2_ due to selective He^+^ irradiation was investigated by utilizing (exposing) a standard microscope white light source. Figure 6a shows a log plot for the current-voltage (I_DS_ vs V_DS_) curves at zero gate bias of the homo-junction without (black) and with (red) white light exposure. Significant improvement in channel current is observed due to the built-in electric potential at the junction. A photovoltaic effect with open circuit voltage of 220 mV is observed (see Fig. 6b), which is comparable with the lateral homo-junction^9^ and hetero-junction^11^,^12^ in mono and few-layer WSe_2_ and MoS_2_ devices, respectively. No significant photovoltaic effect is observed in the pristine WSe_2_ device without He^+^ irradiation (see the Supplementary Information Fig S12). This confirms the presence of homo-junction in few-layer WSe_2_ due to the selective defect introduction from irradiation with He^+^.

**Figure 7:**
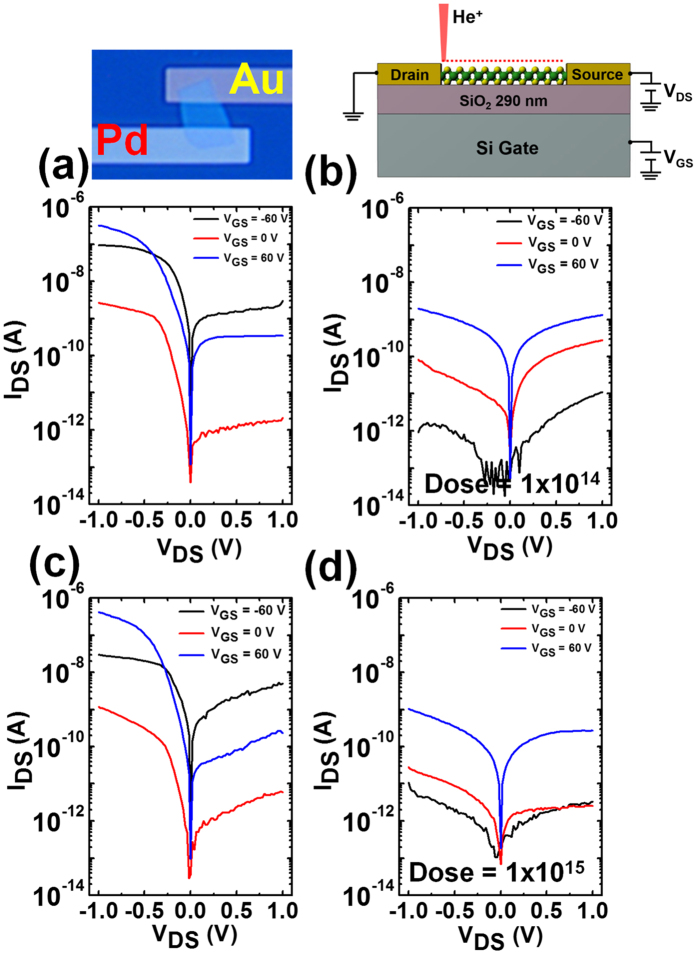
The semi-log plot of output characteristics (I_DS_ vs V_DS_) of asymmetric electrodes (Pd in one side and Ti/Au in other side) of few-layer WSe_2_ devices, before (left panel (**a**,**c**)) and after (right panel (**b**,**d**)) He^+^ irradiations at two different doses (1 × 10^14^ and 1 × 10^15 ^He^+^/cm^2^, respectively). Preferential hole injection in the WSe_2_ channel is clearly seen from the asymmetric nature of I_DS_–V_DS_ curves (**a**,**c**) due to the ohmic contact between the Pd electrode and valence band of WSe_2_ flake and a possible Schottky barrier at Ti/Au contact. The hole transport is still significantly suppressed compared to electron transport (Fig. b,d) on WSe_2_ channel after the He^+^ irradiation. The images on the top of the figure depict an optical micrograph (left) and schematic (right) of the device structure studied.

To further investigate the effect of He^+^ irradiation on hole transport in few-layer WSe_2_, we also fabricated an asymmetric electrode (Pd in one and Ti/Au in the other) device with the favorable energy band alignment for hole collection by minimizing the Schottky barrier between the valence band of the WSe_2_ and fermi level of Pd metal electrode. We carried out the electrical transport measurements before (Fig. 7a,c) and after (Fig. 7b,d) He^+^ irradiation for two different ion doses (1 × 10^14 ^ions/cm^2^ – upper panel and 1 × 10^15 ^ions/cm^2^ – lower panel, respectively). Preferential hole injection in the WSe_2_ channel is clearly observed in the asymmetric nature of I_DS_–V_DS_ curves (see Fig. 7a,c) due to the Ohmic contact between the Pd electrode and valence band of WSe_2_ flake relative to the apparently small Schottky barrier for the Ti/Au contact. The hole transport is still significantly suppressed compared to electron transport (see Fig. 7b,d) in the WSe_2_ channel after He^+^ irradiation. For instance, the device ON current decreased from 30 nA (pristine device) to 10 pA at V_DS_ = −1 V and V_GS_ = −60 V, due to He^+^ irradiation at the dose of 1 × 10^15 ^ions/cm^2^.

## Discussion

In summary, we report the effects of focused helium-ion beam irradiation on opto-electronic properties of few-layer WSe_2_ devices. Precise defects were selectively introduced in mechanically exfoliated few-layer WSe_2_ by controlled dose of He^+^ irradiation, and its effects on structural, optical and electrical properties were investigated via STEM, Raman spectroscopy, and transport measurements. With increasing dose, point defects and local disorder of WSe_2_ flakes were observed, thereby tuning the electrical transport of the material, and allowing control over semiconductor-insulator-metal like transitions with more than six order change in resistivity. Hole transport in WSe_2_ was significantly suppressed compared to electron transport for the same dose of He^+^ irradiation. This presents the unprecedented opportunity to create direct –write lateral junctions in the materials. By selective He^+^ irradiation, we demonstrate a lateral homo-junction, like a conventional p-n junction, which constitute an important advance towards two dimensional opto-electronic devices.

## Methods

### Helium ion irradiation

Helium ion exposures were performed with a Zeiss ORION NanoFab He/Ne ion microscope. An accelerating voltage of 25 keV was used for all exposures. Beam currents were varied from 0.3–6.0 pA in order to enable a large range of exposure doses (1 × 10^12^–1 × 10^17 ^ion/cm^2^). All patterns in this study were exposed with a constant 1 μs dwell time, whereas the pixel spacing was varied with the desired dose. For low dose exposures (<1 × 10^14 ^ions/cm^2^), larger pixel spacing (4–40 nm) with beam defocus was utilized to supply a uniform dose to the pattering area. For higher doses (>1 × 10^14 ^ion/cm^2^), a pixel spacing of 2 nm was used. Patterns were generated using Fibics NPVE pattern generating software and hardware scan controller.

### WSe_2_ device fabrication and characterization

Polycrystalline WSe_2_ was synthesized from a stoichiometric mixture of W (Alfa-Aesar, 99.999%) and Se (Alfa-Aesar, 99.999%) powders. The starting materials were sealed in silica tubes under vacuum, and then slowly heated to 900 °C. The ampoules remained at 900 °C for seven days, and then were allowed to furnace cool to room temperature. Single crystals of WSe_2_ were then grown using the polycrystals as starting material and iodine as a transport agent. The silica tubes containing phase-pure powder and iodine were sealed under vacuum and placed in a tube furnace with a 50 °C temperature gradient from the hotter end of the tube containing the charge (1050 °C) to the colder end where growth occurs (1000 °C). The iodine concentration within the tube was ~17.5 mg/cm^3^. Crystals in the form of shiny silver plates with typical size 5 × 5 × 0.1 mm^3^ grew over the course of 5 days. WSe_2_ flakes were exfoliated onto SiO_2_ (290 nm)/Si (heavily doped Si which also serves as a bottom gate electrode) substrate from a bulk single crystal by the ‘Scotch tape’ micromechanical cleavage technique and were identified by their optical contrast. The thicknesses of the exfoliated WSe_2_ flakes were measured using Atomic Force Microscope (AFM). Kelvin probe force microscopy (KPFM) measurements were performed using an Asylum Research Cypher AFM with a Pt-Ir coated cantilever. Standard e-beam lithography followed by e-beam evaporation was employed to create the source/drain electrodes for electrical measurements. The contacts consisted of Ti/Au (5/30 nm) metals deposited and subsequently patterned via a lift-off process. The fabricated devices were subjected to He^+^ exposures with different doses ranging from 1 × 10^12^ to 1 × 10^17 ^ions/cm^2^. The electrical characteristics of the fabricated WSe_2_ devices before and after the He^+^ exposure were measured using an Agilent semiconductor parametric analyzer (Agilent Tech B1500 A).

### Raman spectroscopy

Raman spectroscopy and mapping were performed in a Renishaw inVia micro-Raman system using a 532 nm excitation laser. A 100X magnification objective was used for spectral acquisition with a 5 second acquisition time. Maps were generated using a 0.6–1 μm step size. Data analysis and maps were constructed with the WIRE v3.4 software.

### Energy dispersive X-ray spectroscopy

Energy dispersive X-ray spectroscopy (EDS) was conducted in a Zeiss MERLIN Scanning Electron Microscope (SEM) equipped with a Bruker EDS system. For the EDS measurements, a map acquisition of the He^+^ irradiated WSe_2_ film was taken over a ~6 × 8 μm area with a 15 min collection time. A beam energy of 4 keV and beam current of 0.7 nA were used to excite the sample and generate the X-ray spectra.

### Microscopy

Atomic resolution images of WSe_2_ were acquired using a Nion UltraSTEM100 scanning transmission electron microscope with fifth-order aberration correction. STEM was operated at 60 kV with a spatial resolution of 1.1 angstrom. High angle annular dark-field (HAADF) Z-contrast images of suspended WSe_2_ were recorded for regions exposed to He^+^ doses of 2 × 10^13^, 1 × 10^15^, 1 × 10^16^, and 1 × 10^17 ^ions/cm^2^. The WSe_2_ flake was exfoliated onto a holey silicon nitride membrane with 2.5 μm holes prior to exposure and imaging. SAED patterns were taken after imaging at the same locations with a Zeiss Libra 200 MC operated at 200 keV.

## Additional Information

**How to cite this article**: Stanford, M. G. *et al.* Focused helium-ion beam irradiation effects on electrical transport properties of few-layer WSe_2_: enabling nanoscale direct write homo-junctions. *Sci. Rep.* **6**, 27276; doi: 10.1038/srep27276 (2016).

## Supplementary Material

Supplementary Information
