# Supplementary material for: Focused helium-ion beam irradiation effects on electrical transport properties of few-layer WSe2: enabling nanoscale direct write homo-junctions
Source: Sci Rep. 2016 Jun 6;6:27276. doi: 10.1038/srep27276 (PMC4893660; doi:10.1038/srep27276)
Supplement: Supplementary Information [file srep27276-s1.pdf]

Supporting Information

Focused helium-ion beam irradiation effects on electrical transport properties of few-layer WSe_2: enabling nanoscale direct write homo-junctions

*Michael G. Stanford[1], Pushpa Raj Pudasaini[1], Alex Belianinov[2], Nick Cross[1], Joo Hyon Noh[1], Michael Koehler[1], David G. Mandrus[1,3], Gerd Duscher[1,3], Adam J. Rondinone[2], Ilia N. Ivanov[2], T. Zac Ward[3], Philip D. Rack[1,2]**

1. Department of Materials Science and Engineering, University of Tennessee, Knoxville, Tennessee 37996, United States
2. Center for Nanophase Materials Sciences, Oak Ridge National Laboratory, Oak Ridge, Tennessee 37831, United States
3. Materials Science and Technology Division, Oak Ridge National Laboratory, Oak Ridge, Tennessee 37831, USA

Raman Spectroscopy

Table S1 lists the Raman peak assignments for few-layer WSe_2. The spectra as a function of various exposure doses can be found in Figure S1. It is clear that He^+ exposure causes a reduction in the E^1_{2g} and A_{1g} peaks, as well all multiplicity peaks. A sharp rise in the LA(M) peak is observed as the He^+ exposure dose is increases. This is indicative of selective sputtering and defect introduction within the WSe_2 flake.

Table S1. Raman peak assignments of few-layer WSe_2.

Peak (cm^{-1})	Assignments[1]
119	LA(M)
138	A_{1g}-LA
238	2LA(M)
250	E^{1}_{2g} (in plane)
258	A_{1g} (out of plane)
362	$2E_{1g}$
373	A_{1g}+LA
395	$2A_{1g}$-LA

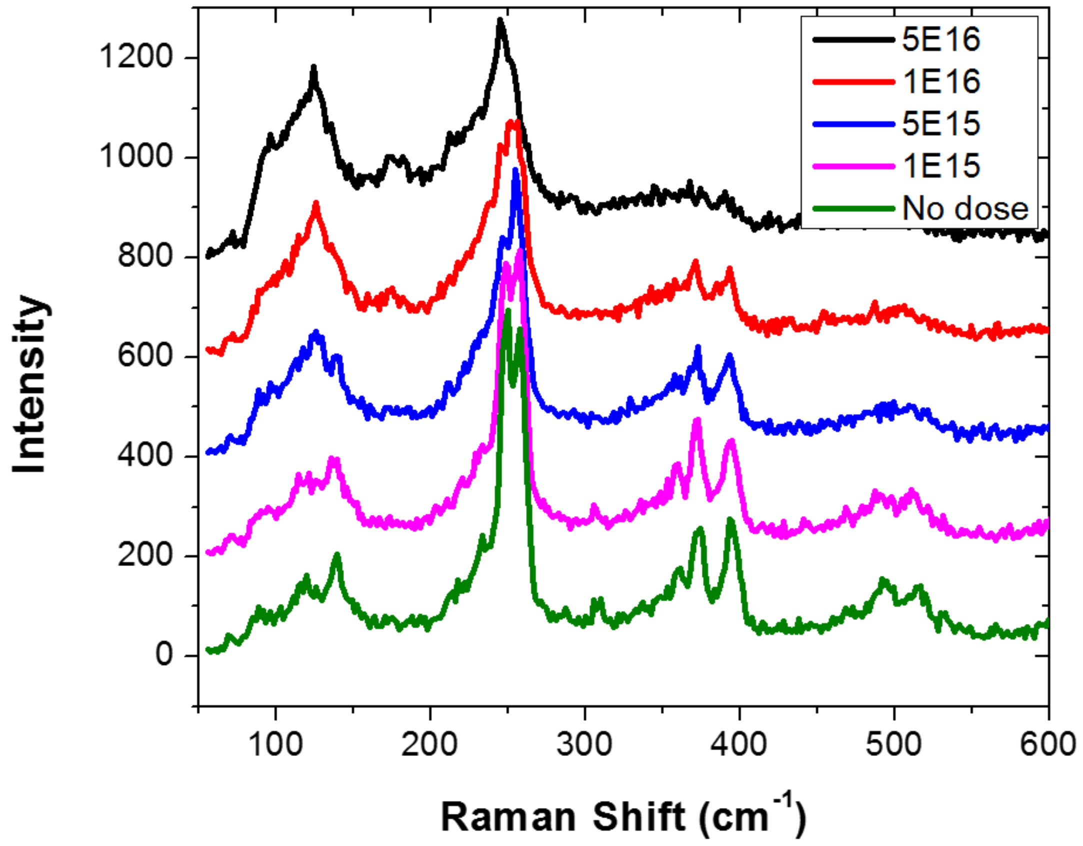

Figure S1. Raman spectra of few-layer WSe_2 at various He^+ exposure doses.

STEM

Figure S2 displays high-resolution HAADF STEM images of suspended WSe_2 that was irradiated with He^+ doses from $2x10^{13}$ – $1x10^{17}$ ions/cm^2. There is a significant increase in disorder with increasing He^+ dose. Clearly, increasing dose introduces greater amounts of disorder and defects into the WSe_2. An indexed SAED pattern is shown in Figure S3. Figure S4 compares the Z-contrast STEM images, Fourier transformations of each respective Z-contrast image, and selected area electron diffraction patterns that correspond to each He^+ dose which was studied. The Fourier transformations of the Z-contrast images agree well with SAED results.

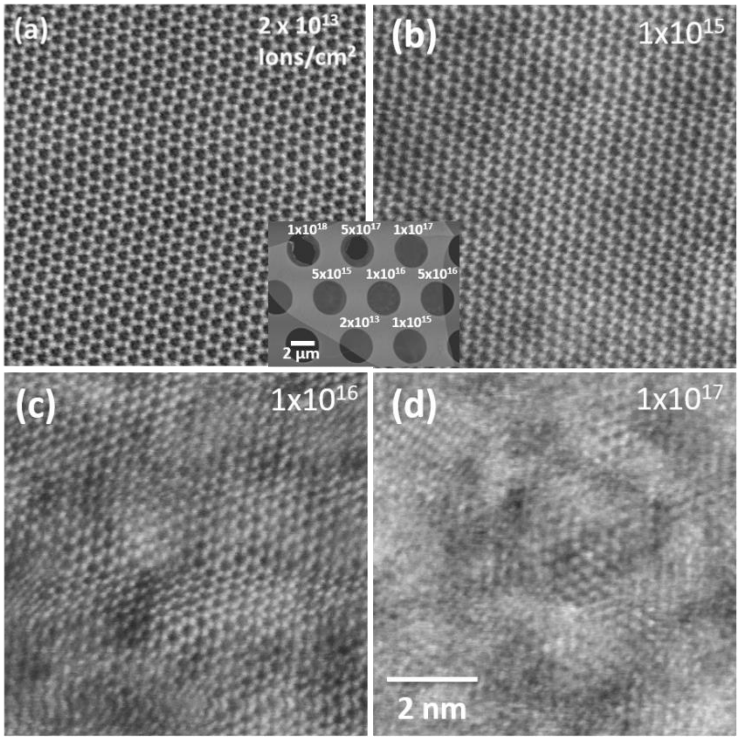

Figure S2. HAADF STEM images of suspended WSe_2 which was irradiated with He^+ at doses of A) $2x10^{13}$, B) $1x10^{15}$, C) $1x10^{16}$, and D) $1x10^{17}$ ions/cm^2. Images have 8 nm field of view.

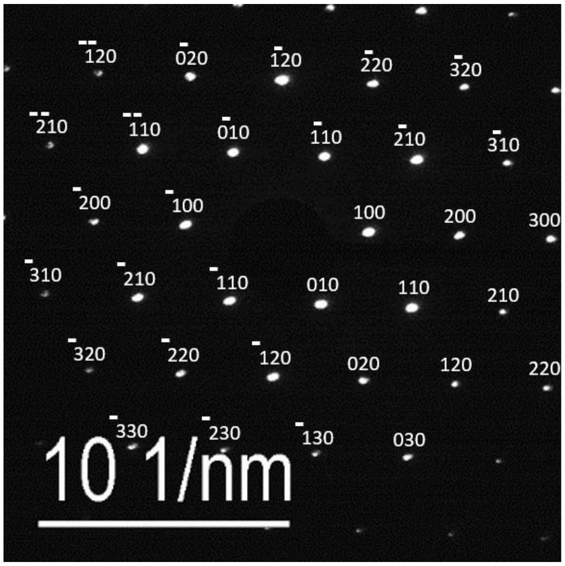

Figure S3. Indexed diffraction pattern taken from WSe_2 flake exposed with a dosage of 2x10^{13} He^{+}/cm^{2}.

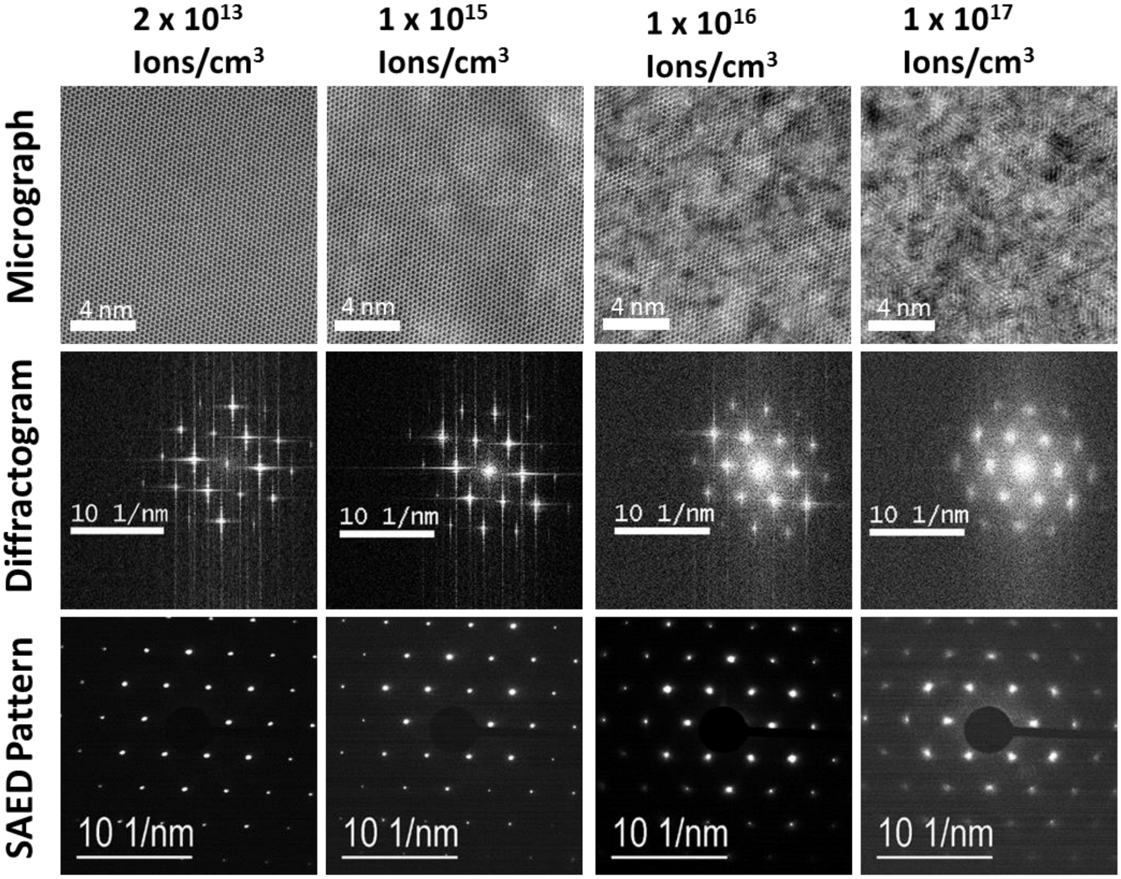

Figure S4. Figure compares the changes in the WSe_2 crystal structure when subjected to varying doses of ion irradiation. This has been done by STEM Z-contrast imaging, Fourier transformations of each respective Z-contrast image, and selected area electron diffraction patterns that correspond to each region. Fourier transformations of the Z-contrast images agree well with SAED results.

EDS

Suspended WSe_2 was irradiated with the 30 keV He^+ beam at various doses ($1x10^{15}$ – $1x10^{18}$ ions/cm^2) and is displayed in Figure S5a. Clearly a dose of greater than $5x10^{17}$ is sufficient to completely sputter away the entire WSe_2 film. Signs of modification of the film are apparent with exposure down to a dose of $5x10^{16}$ ions/cm^2. Chemical composition analysis of the irradiated films were conducted using energy-dispersive X-ray spectroscopy (EDS). In order to qualitatively determine compositional changes in the WSe_2 with He^+ exposure, the relative peak ratios of W - M (1.774 keV) and Se - L (1.379 keV) were compared and reported in Figure S5b. With increasing He^+ exposure, the W composition relative to Se increases. This is consistent with Fox et al.[7], which demonstrates that He^+ irradiation results in the preferential sputtering of chalcogens (S) in MoS_2 films. The chalcogen is preferentially sputtered since its atomic mass is nearly 3x less than that of W, and momentum exchange with the energetic He^+ is sufficient to eject Se atoms. The preferential sputtering in essence enables selective doping of the WSe_2, by creating direct-write chalcogen deficient regions.

Raw EDS spectra for WSe_2 irradiated with He^+ at doses from 1×10^{12} – 3×10^{17} ions/cm^2 are shown in Figure S6a. The spectra were collected by taking an area scan over the entire region exposed by the He^+ beam. At a dose of 5×10^{17} ions/cm^2 the He^+ irradiation completely sputters away the WSe_2 films. The reduction in overall intensity of the EDS spectra with increasing dose

is due to material removal, thus making the EDS response weaker. The W/Se intensity ratio increases with increasing He^+ dose due to preferentially sputtering of Se as shown in Figure S5b. Figures S6b-e show EDS area maps from which the raw spectra were acquired.

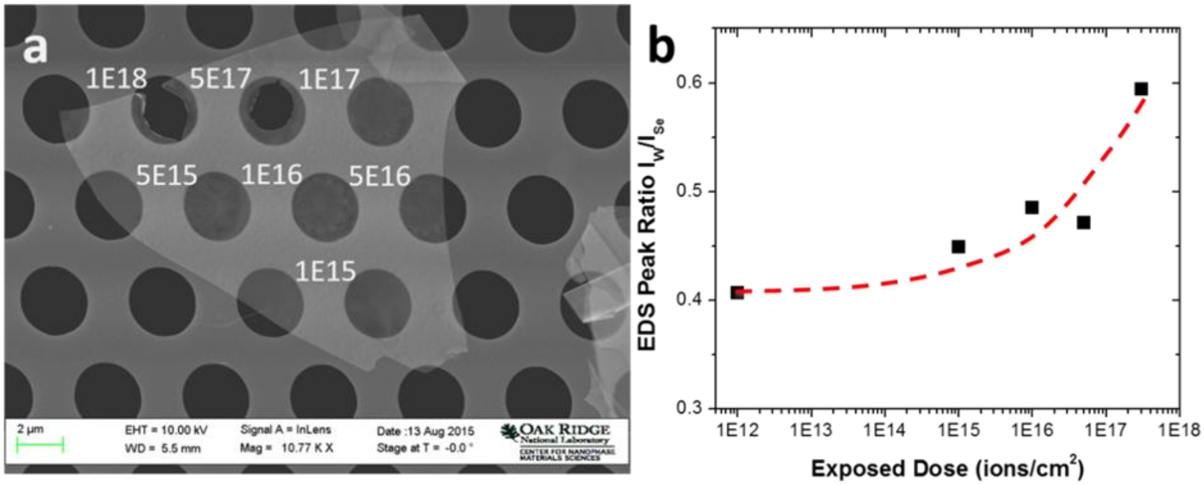

Figure S5. (a) SEM image of a single layer WSe_2 flake on a holey silicon nitride membrane. Inset doses denote the dose applied to each suspended region with units of ions/cm^2. (b) Plot of the ratio of the relative peaks intensities of W – M and Se – L as a function of He^+ dose.

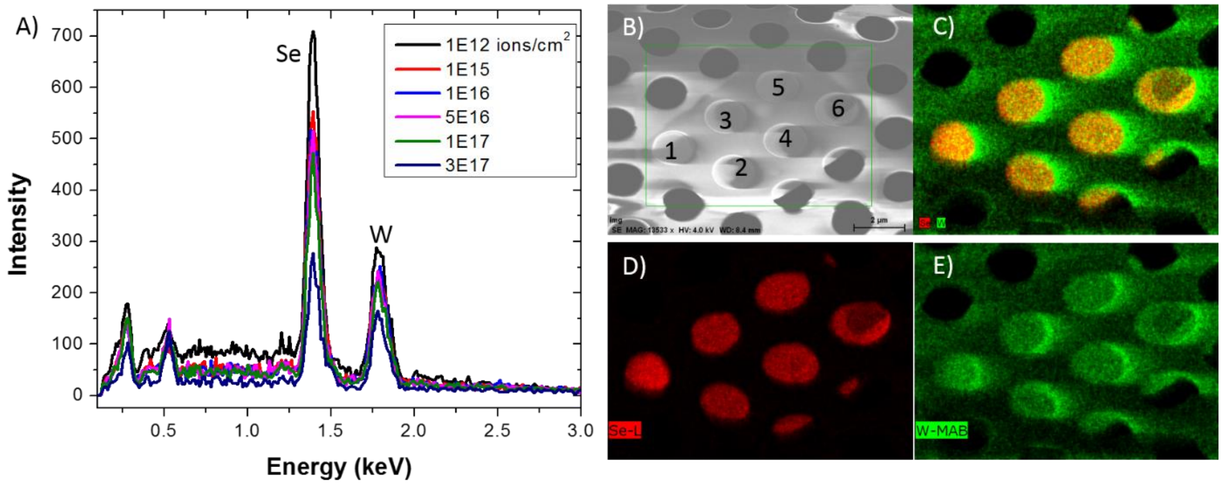

Figure S6. A) Raw EDS spectra for suspended WSe_2 exposed with He^+ of various doses. B) SEM image of the exposed suspended flake on silicon nitride where regions 1-6 were exposed with doses of 1E12, 1E17, 5E16, 1E16, 1E15, and 3E17 respectively. C-E) EDS maps of Se + W, only Se, and only W respectively.

Hysteresis on transfer curves

The transfer curves (I_{DS} vs V_{GS}) of all few layers WSe_2 FET device were collected reversibly (double sweep) with the gate voltage ranging from -60 V to +60 V, at different source-drain voltages (V_{DS}). A small hysteresis on the measured channel current (I_{DS}) was observed for the device before and after the He+ ions irradiation. A typical hysteresis collected in one of the device studied is shown below, however, transfers curves with a single voltage sweep were reported throughout the manuscript for clarity.

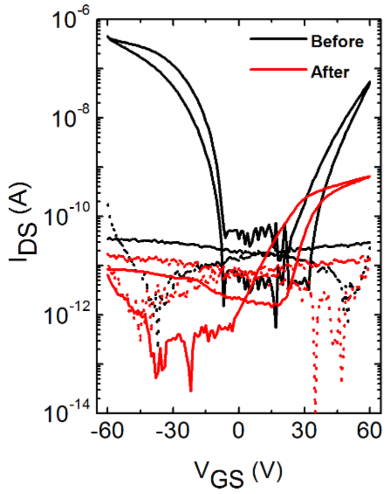

Figure S7. The typical hysteresis in measured I_{DS} vs V_{GS} curves at V_{DS} = 0.1 V, before (black) and after (red) He^+ irradiation at the dose of 1.0 x 10^{15} ions/cm^2. The corresponding leakage currents (doted lines) are also plotted in the same graph.

Mobility Thickness Dependence

Field effect mobility was extracted from FETs fabricated from various thicknesses of exfoliated WSe_2 and reported in Figure S8. The maximum field effect mobility of 64.13 cm^2/V.s for hole conduction for a device with a 9 nm WSe_2 thickness was recorded. At greater thicknesses, the field effect mobility is significantly reduced.

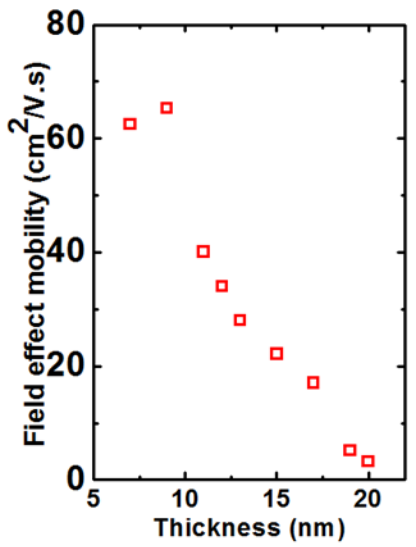

Figure S8. Field effect hole mobility extracted from the transfer characteristic curves for few-layer WSe_2 devices as a function of flake thickness.

He^+ dose effect on electrical transport properties of few layers WSe_2 as a function of thickness

The He^+ irradiation effect as a function of WSe_2 film thickness was also studied at a common dose of 1 x 10^{15} ions/cm^2. I-V measurements reveal that irrespective to the WSe_2 channel thickness, both hole and electron conductivity were significantly suppressed (hole conduction decreased more than electron conduction) and it shows slightly n-type behavior (increase in channel current with the increase in gate voltage). Prior to the He^+ exposure, the ON state currents for both electron (at + 60 V gate bias) and hole (at -60 V gate bias) were on the order of 1 µA (normalized to channel W/L ratio). The field effect electron mobility in one of the pristine WSe_2 FET devices prior to the He^+ irradiation was measured to be 32.80 cm^2/V.s, which decreased to 0.08 cm^2/V.s after the He^+ irradiation at a dose of 1 x 10 ^{15} ions/cm^2. It is worth

noting that a full channel exposure at this dose (1x10^{15} He^+/cm^2) results in insulating behavior of the film. Although the electron mobility was noticeable decreased, electron conduction after irradiation is still far greater than hole conduction.

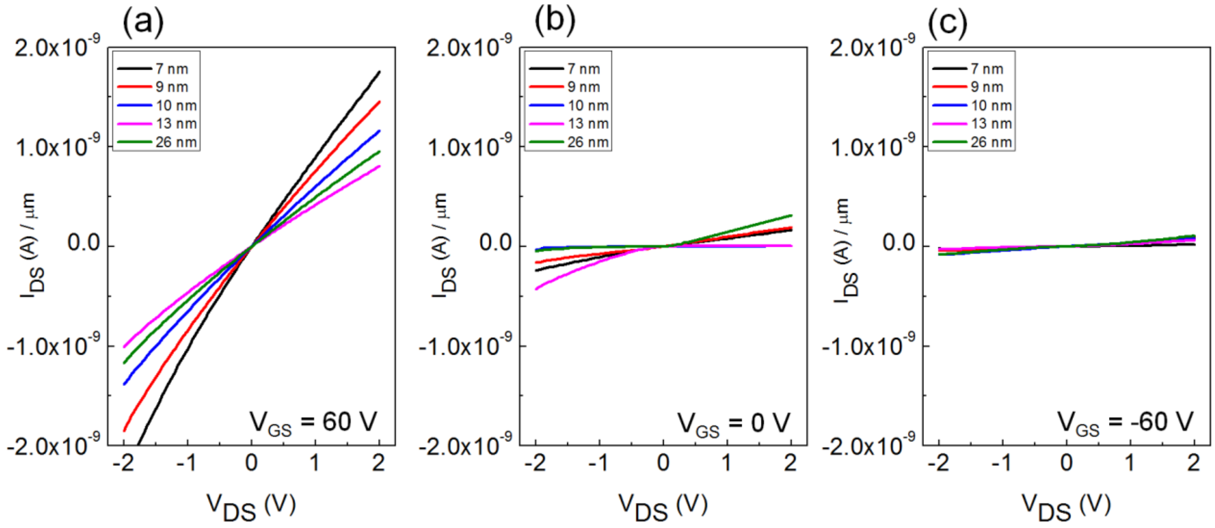

Figure S9. Measured I_{DS} vs V_{DS} normalized to channel W/L ratio, for the different thickness of WSe_2 flakes at three different gate voltages, a) V_{GS} = 60 V, b) V_{GS} = 0 V and c) V_{GS} = -60 V. All flakes were exposed in the channel region with a dose of 1x10^{15} He^+/cm^2.

EnvizION ion-solid Monte Carlo simulation

EnvizION Monte Carlo simulations[2] were conducted in order to determine atom displacements created by 25 keV energetic He^+ in WSe_2 (ignoring channeling effects). Figure S10 shows cross-sections of WSe_2 films of varying thickness which were exposed to a dose of 1x10^{15} He^+/cm^2. Green pixels represent Se atoms, blue pixels represent W atoms, light green pixels represent

displaced Se atoms, light blue pixels represent displaced W atoms, and red pixels represent unfilled vacancies created by sputtering events. The distribution of defect sites are largely uniform over the exposed regions and appear to be independent of film thickness, since the films are much thinner than the penetration depth of 25 keV He^+ in WSe_2. Table S2 list the sputter yield and Se/W sputter ratio at the three WSe_2 thicknesses which were simulated. The simulations support experimental findings which indicate the Se is preferentially sputtered in comparison to W under He^+ irradiation.

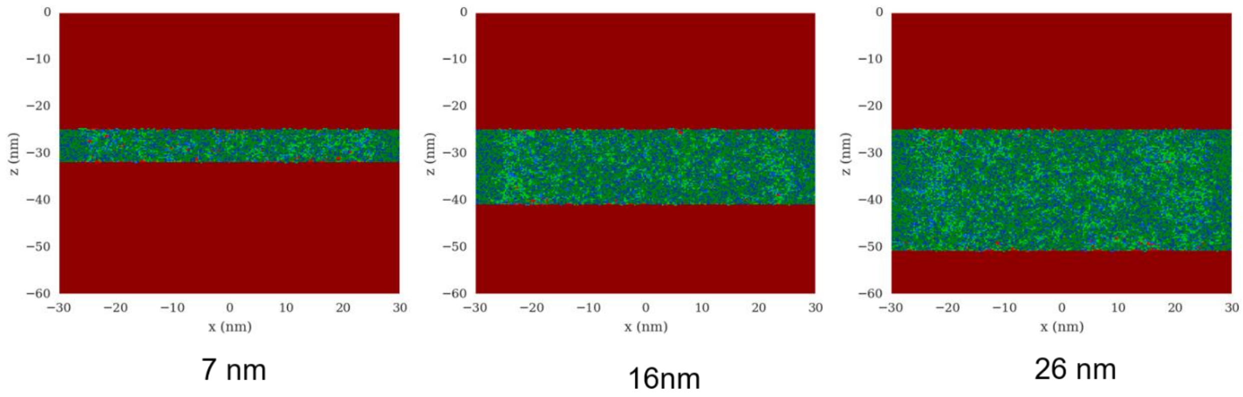

Figure S10. Using our EnvizION[2] ion-solid Monte Carlo simulation we simulated varying thickness WSe_2 films (7, 16, and 26 nm) which were exposed with 25 keV He^+ to a dose of $1x10^{15}$ He^+/cm^2. In this simulation, the WSe_2 is assumed to be amorphous with the stoichiometric W/Se ratio of 1/2 and using the bulk properties of crystalline WSe_2. The He^+ was simulated as a 25 nm cylindrical beam. Green pixels represent Se atoms, blue pixels represent W atoms, light green pixels represent displaced Se atoms, light blue pixels represent displaced W atoms, and red pixels represent unfilled vacancies created by sputtering events.

Table S2. EnvizION Monte Carlo simulation sputter yields and Se/W sputter ratio for WSe_2 of varying thicknesses.

Thickness (nm)	Yield (atoms/ion)	Ratio (Se/W)
7	0.10	3.90
16	0.11	3.4
26	0.11	3.5

Device Aging

Aging effects on a device exposed with a dose of 1x10^{14} ions/cm^2 was measured over the course of 30 days and displayed in Figure S11. The transistor ON current, with a V_{DS} = 1.1 V and V_{GS} = 60 V, remained constant during this time period. The lack of ON current recovery suggest that stable defects were formed within the WSe_2 flake and are not simply due to fixed oxide positive charge induced in the underlying substrate, which exhibits a recoverable ON current>.

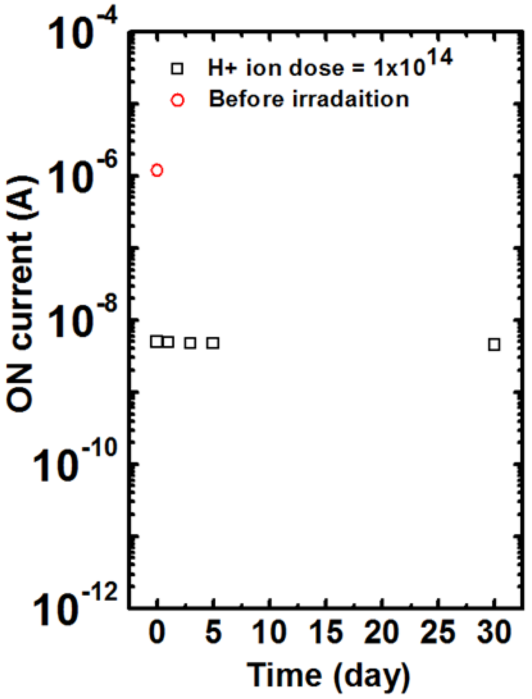

Figure S11. Time dependence of transistor ON current at V_{DS} = 1.1V and V_{GS} = 60 V, for few-layer WSe_2 device irradiated with He^+ ion dose at $1x10^{14}$ up to 30 days. No recovery on transistor ON currents has been observed by exposing the irradiated sample in ambient conditions. The red open circle represents the channel current prior to He^+ exposure.

Photoresponse in unexposed device

Figure S12 shows the output characteristics of pristine WSe_2 devices under dark and light conditions. Unlike the devices which have a direct-write He^+ exposed junction (Figure 6a), there is no open circuit voltage (V_{OC}) under light conditions. Currents are slightly greater with light conditions due additional excited charge carriers, however there is no significant photovoltaic effect.

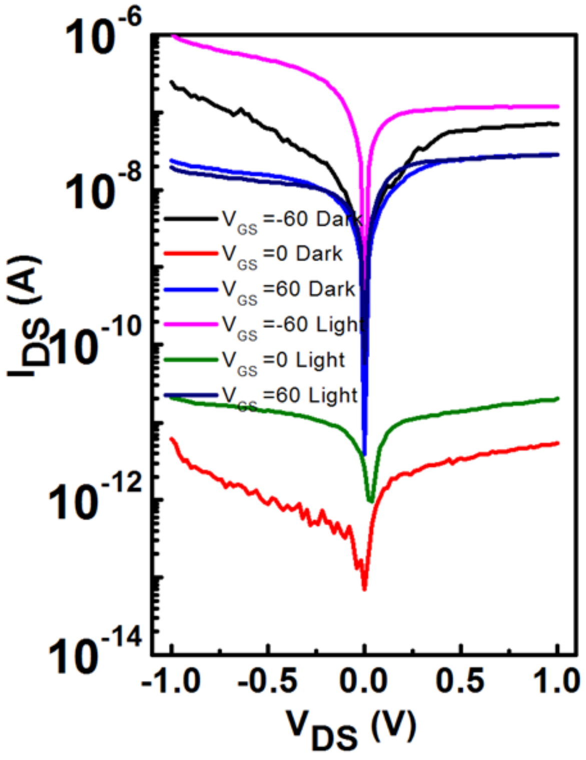

Figure S12. Photo-response of one of the pristine few-layer WSe_2 device (without He+ irradiation) at various gate potentials. No significant photovoltaic effect has been observed in few-layer WSe_2 device.

References

(1) Li, H.; Lu, G.; Wang, Y.; Yin, Z.; Cong, C.; He, Q.; Wang, L.; Ding, F.; Yu, T.; Zhang, H. Mechanical Exfoliation and Characterization of Single- and Few-Layer Nanosheets of WSe2, TaS2, and TaSe2. *Small* **2013**, *9*, 1974–1981.

(2) Timilsina, R.; Tan, S.; Livengood, R.; Rack, P. D. Monte Carlo Simulations of Nanoscale Focused Neon Ion Beam Sputtering of Copper: Elucidating Resolution Limits and Sub-Surface Damage. *Nanotechnology* **2014**, *25*, 485704.

(3) Kim, T.-Y.; Cho, K.; Park, W.; Park, J.; Song, Y.; Hong, S.; Hong, W.-K.; Lee, T. Irradiation Effects of High-Energy Proton Beams on MoS2 Field Effect Transistors. *ACS Nano* **2014**, *8*, 2774–2781.
